# Compound Heterozygous Complete Loss-of-Function *SPINK1* Variants as a Novel Cause of Severe Infantile Isolated Exocrine Pancreatic Insufficiency

**DOI:** 10.3390/genes16090998

**Published:** 2025-08-25

**Authors:** Emmanuelle Masson, Marc Wangermez, David Tougeron, Vinciane Rebours, Claude Férec, Jian-Min Chen

**Affiliations:** 1 Inserm, Univ Brest, EFS, UMR 1078, GGB, 29200 Brest, France; emmanuelle1.masson@chu-brest.fr (E.M.); claude.ferec@gmail.com (C.F.); 2 Service de Génétique Médicale et de Biologie de la Reproduction, CHU Brest, 29200 Brest, France; 3 Gastroenterology Department, Poitiers University Hospital, 86000 Poitiers, France; marc.wangermez@chu-poitiers.fr (M.W.); david.tougeron@chu-poitiers.fr (D.T.); 4 Pancreatology and Digestive Oncology Department, Beaujon Hospital, APHP—Clichy, Université Paris Cité, 75006 Paris, France; vinciane.rebours@aphp.fr

**Keywords:** complex genomic rearrangement, compound heterozygosity, deletion variant, loss-of-function allele, rare pediatric disease, missing heritability, serial replication slippage, severe infantile isolated exocrine pancreatic insufficiency, SPINK1 protein, translesion synthesis

## Abstract

Background/Objectives: While complete loss-of-function (LoF) *SPINK1* variants in the simple heterozygous state cause chronic pancreatitis, biallelic complete LoF variants result in a rare pediatric disorder termed severe infantile isolated exocrine pancreatic insufficiency (SIIEPI). To date, only two individuals with a null *SPINK1* genotype have been reported—one homozygous for a whole-gene deletion and the other for an *Alu* insertion in the 3′ untranslated region. Here, we report the genetic basis of a third SIIEPI case, presenting in early infancy with severe exocrine pancreatic insufficiency and diffuse pancreatic lipomatosis. Methods: Targeted next-generation sequencing (NGS) was used to analyze the entire coding region and exon–intron boundaries of the *SPINK1* gene. Copy number variant (CNV) analysis was performed with SeqNext, based on normalized amplicon coverage. Results: The proband harbored compound heterozygous complete LoF *SPINK1* variants. One was the known NM_001379610.1:c.180_181del (p.(Cys61PhefsTer2)), inherited from the father. The second, initially detected as an exon 2 deletion and confirmed by quantitative fluorescent multiplex PCR (QFM-PCR), was further characterized by long-range PCR as a complex rearrangement comprising a 1185 bp deletion removing exon 2, a 118 bp templated insertion followed by a non-templated nucleotide, and an 8 bp deletion. The mutational signature is consistent with serial replication slippage or template switching involving translesion synthesis. This maternally inherited variant has not been previously reported. Conclusions: This study expands the mutational spectrum of *SPINK1*-related SIIEPI and suggests that this distinct pediatric disorder may be under recognized in clinical practice.

## 1. Introduction

The *SPINK1* gene (serine peptidase inhibitor, Kazal type 1; OMIM #167790), located on chromosome 5q32, encodes the pancreatic secretory trypsin inhibitor (PSTI) [1,2]. PSTI, produced mainly by pancreatic acinar cells, has long been thought to protect the pancreas by inhibiting prematurely activated trypsin [3,4]. The association of loss-of-function (LoF) *SPINK1* variants with chronic pancreatitis (CP; OMIM #167800) [5] supports this protective role. Mouse studies further reinforce this, showing that *SPINK1* overexpression reduces pancreatitis severity [6,7,8,9]. Together with gain-of-function missense and copy number variants in the *PRSS1* gene (encoding cationic trypsinogen; OMIM #276000) [10,11] and LoF variants in the *CTRC* gene (encoding chymotrypsin C, which degrades trypsin [12]; OMIM #601405) [13,14], these findings have established a trypsin-dependent pathway in CP pathogenesis [15,16]. For a recent review on *SPINK1*, see Wang et al. [17].

To date, numerous LoF *SPINK1* variants have been reported [18]. While simple heterozygous LoF *SPINK1* variants are well recognized to predispose to or cause CP, biallelic variants resulting in complete LoF of *SPINK1* lead to a severe pediatric disorder termed severe infantile isolated exocrine pancreatic insufficiency (SIIEPI) [19]. Only two SIIEPI cases have been described. One patient was homozygous for a whole-gene deletion, which resulted in a null genotype. The other carried a homozygous *Alu* insertion in the 3′ untranslated region [19], which silences *SPINK1* expression by promoting RNA secondary structure formation with pre-existing deep intronic *Alu* elements [20]. Consistent with these human findings, *Spink1*-deficient mice died perinatally due to massive necrosis of pancreatic acinar cells [21,22].

Here, we report compound heterozygosity for complete LoF *SPINK1* variants, with a known 2 bp frameshift deletion [23] on one allele and a previously unreported complex rearrangement on the other, as an additional genetic basis for *SPINK1* deficiency in humans.

## 2. Materials and Methods

### 2.1. Ethics Statement

This study was conducted in accordance with the Declaration of Helsinki and was approved by the Ethics Committee of Brest University Hospital (No. 3.347-A; approval date: 3 June 2003). Written informed consent was obtained from all participants.

### 2.2. Reference Sequences, Variant Nomenclature, and Novel Variant Deposition

As in our previous studies [20,24], NM_001379610.1 and NG_008356.2 were used as the reference *SPINK1* mRNA and genomic sequences, respectively. The novel *SPINK1* variant reported in this study was named in principle according to the Human Genome Variation Society (HGVS) guidelines (https://hgvs-nomenclature.org/stable/, accessed on 18 June 2025) [25] and is deposited in GenBank (https://www.ncbi.nlm.nih.gov/genbank/, accessed on 12 June 2025; accession PV784948).

### 2.3. Variant Detection

Targeted next-generation sequencing (NGS) was performed on the proband to analyze the entire coding region and exon–intron boundaries of the *SPINK1* gene. PCR amplification was carried out using a 48.48 Fluidigm Access Array (Fluidigm, Les Ulis, France) with the FastStart™ High Fidelity PCR System Kit (Sigma Aldrich Chimie S.a.r.l., Saint-Quentin-Fallavier, France). The targeted DNA sequencing library was prepared following Illumina’s standard protocol and sequenced on the MiniSeq system (Illumina, San Diego, CA, USA). Primer sequences and NGS conditions are available upon request from the first author (E.M.). NGS data were analyzed using the SeqNext application (JSI Medical Systems, Ettenheim, Germany).

Copy number variant (CNV) analysis was also performed using the SeqNext application, based on normalized amplicon coverage. For each individual, the read depth of each target amplicon was first normalized against two internal control amplicons. The same normalization was applied to two wild-type (WT) control DNA samples. For each target amplicon, the normalized average coverage from the WT controls was calculated and used as the reference for computing the relative coverage in the study individual. Any amplicon with relative coverage below 75% or above 125% of the control average was classified as a deletion or duplication, respectively.

Targeted NGS identified two variants in the proband. The first was the previously reported NM_001379610.1(*SPINK1*):c.180_181del variant [23], which was confirmed by Sanger sequencing. For this, PCR amplification was performed using the HotStarTaq Master Mix kit (QIAGEN, Courtaboeuf, France) with 50 ng of genomic DNA in a 10 µL reaction containing primers forward (5′-CAATCACAGTTATTCCCCAGAG-3′) and reverse (5′-CGGGGTGAGATTCATATTATCAG-3′). After initial denaturation at 95 °C for 15 min, the PCR program consisted of 40 cycles of 94 °C for 30 s, 60 °C for 30 s, and 72 °C for 45 s. The 295 bp product was purified with Illustra™ ExoProStar™ (Dominique Dutscher, Issy-les-Moulineaux, France) and sequenced using the BigDye™ Terminator v1.1 Cycle Sequencing Kit (Thermo Fisher Scientific, Illkirch, France) on a 3500 Dx Genetic Analyzer (Thermo Fisher Scientific). Data were analyzed in SeqPatient (v.4.2.2; JSI Medical Systems, Germany).

The second *SPINK1* variant, initially flagged by targeted NGS as an exon 2 deletion, was confirmed by quantitative fluorescent multiplex PCR (QFM-PCR) as previously described [26]. Breakpoint mapping was performed by long-range PCR using the Takara LA Taq with GC Buffer (Takara Bio, Saint-Germain-en-Laye, France) with 50 ng of genomic DNA in a 20 µL reaction containing primers forward (5′-GCCTTGCTGCCATCTGCCA-3′; promoter) and reverse (5′-CGGGGTGAGATTCATATTATCAG-3′, intron 3). After initial denaturation at 94 °C for 1 min, the program consisted of 14 cycles of 94 °C for 20 s and 60 °C for 6 min, followed by 16 cycles of 94 °C for 20 s and 62 °C for 6 min. PCR products (WT band: 3745 bp) were visualized on 1.0% agarose gel; and the patient-specific band (~3.0 kb) was purified using the NucleoSpin Gel and PCR Clean-up Kit (Macherey-Nagel, Düren, Germany). Breakpoints were determined by direct sequencing with BigDye™ Terminator v1.1 Cycle Sequencing Kit and walking primers spaced approximately 500 bp apart. Data were analyzed in SeqPatient.

Parental samples were analyzed to establish the inheritance pattern of both variants.

### 2.4. Public Databases and Online Tools

The Genome Aggregation Database (gnomAD; https://gnomad.broadinstitute.org/, accessed on 12 June 2025) [27], version 4.1.0 or SVs v4.1.0 as appropriate, was used to examine global population allele frequencies of the *SPINK1* variants reported in the proband. The ClinVar database (https://www.ncbi.nlm.nih.gov/clinvar/, accessed on 12 June 2025) [28] was queried to determine whether the novel *SPINK1* variant had been registered. The non-B DNA Motif Search Tool (nBMST; https://nonb-abcc.ncifcrf.gov/apps/nBMST/default/, accessed on 12 June 2025) [29] was used to identify potential non-B DNA motifs that may underlie the formation of the novel *SPINK1* variant.

## 3. Results

### 3.1. The Proband Exhibiting a Phenotype Consistent with SIIEPI

**Figure 1:**
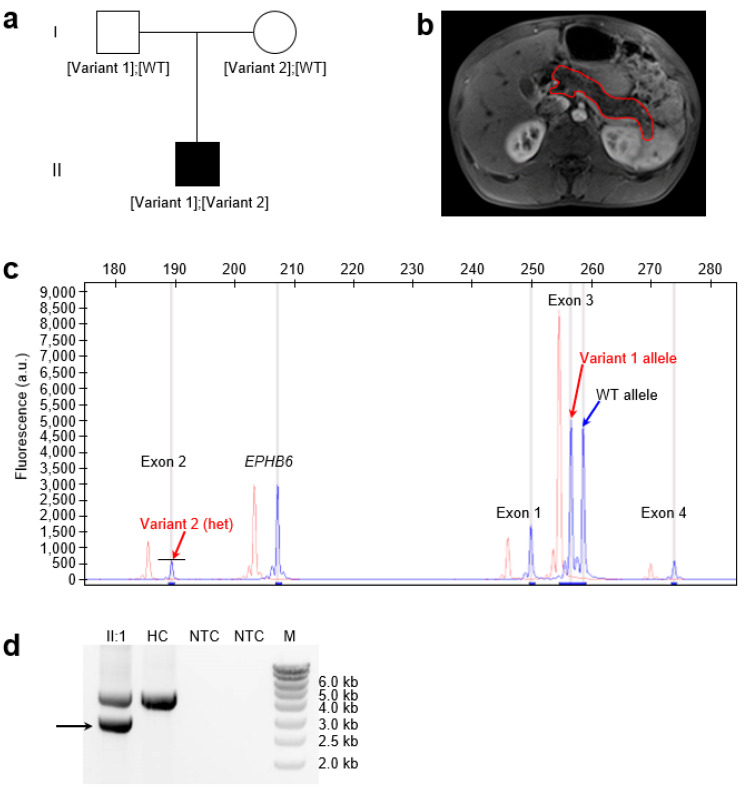
Identification of compound heterozygous *SPINK1* variants in the proband. (**a**) Family pedigree showing inheritance of the two *SPINK1* variants. Variant 1: known c.180_181del; Variant 2: novel complex rearrangement involving the deletion of exon 2. Neither parent had severe infantile isolated exocrine pancreatic insufficiency (SIIEPI), but it is unknown whether they had a history of chronic pancreatitis. (**b**) Axial T2-weighted magnetic resonance image of the abdomen (Dixon in-phase sequence) at age 4. The pancreas, outlined in red, appears normal in size but shows diffusely increased signal intensity, consistent with fatty infiltration (pancreatic lipomatosis). No focal mass, nodules, or ductal dilation is present. The kidneys are normal; portions of the liver and spleen are also visible. (**c**) Detection of both variants in the proband (in blue) by quantitative fluorescent multiplex PCR (QFM-PCR). Control peaks (in red) were intentionally shifted 4 bp to the left. Two distinct peaks indicate the heterozygous c.180_181del variant (Variant 1), corresponding to the WT and deleted alleles. In contrast, the exon 2 deletion (Variant 2) appears as a reduced peak height, consistent with heterozygous loss of the corresponding amplicon. *EPHB6*, internal control amplicon; a.u., arbitrary units; het, heterozygous; WT, wild-type. (**d**) Gel electrophoresis showing the proband-specific long-range PCR product (~3.0 kb; arrow). P, proband; HC, healthy control; NTC, no template control; M, DNA marker. See Supplementary Figure S3 for the uncropped gel image.

The proband, a 16-year-old boy of Senegalese origin, first developed fatty stools at 10 days of age (Figure 1a). Between 5 and 11 months of life, his growth slowed, with reduced weight gain. Occasional abdominal pain was reported, but there were no respiratory symptoms. At 4 years of age, a diagnosis of severe pancreatic exocrine insufficiency was made based on clinical steatorrhea. The diagnosis was confirmed by a fecal elastase concentration of 0.1 μg/g (normal > 200 μg/g). Magnetic resonance imaging showed a normal-sized pancreas with diffuse lipomatosis (Figure 1b), and normal morphology and size of the liver, kidneys, spleen, and gallbladder. The patient was treated with pancreatic enzyme replacement therapy, which led to normalization of growth.

Overall, the clinical presentation of the proband closely resembled that of previously reported SIIEPI cases [19]. No similar cases were identified within the family. It was unknown whether either of the proband’s parents had a history of CP.

### 3.2. Identification of Compound Heterozygous Complete LoF SPINK1 Variants in the Proband

In the proband, targeted NGS revealed two heterozygous variants in *SPINK1*. The first was a 2 bp deletion in exon 3, NM_001379610.1:c.180_181del (p.(Cys61PhefsTer2)), previously reported in the simple heterozygous state in two unrelated individuals with CP [23]. This variant was visualized on QFM-PCR as two distinct, size-separated fluorescent peaks corresponding to the WT and variant alleles (Figure 1c) and confirmed by Sanger sequencing (Supplementary Figure S1). Parental testing indicated paternal inheritance (Figure 1a). It is extremely rare in the general population, with an allele frequency of 6.201 × 10^−7^ (1/1,612,612) in gnomAD v4.1.0. This frameshift variant is interpreted to cause complete LoF of the affected allele [18], as the truncated protein would lack two of the six cysteine residues essential for forming the three disulfide bonds that stabilize the mature WT pancreatic secretory trypsin inhibitor.

**Figure 2:**
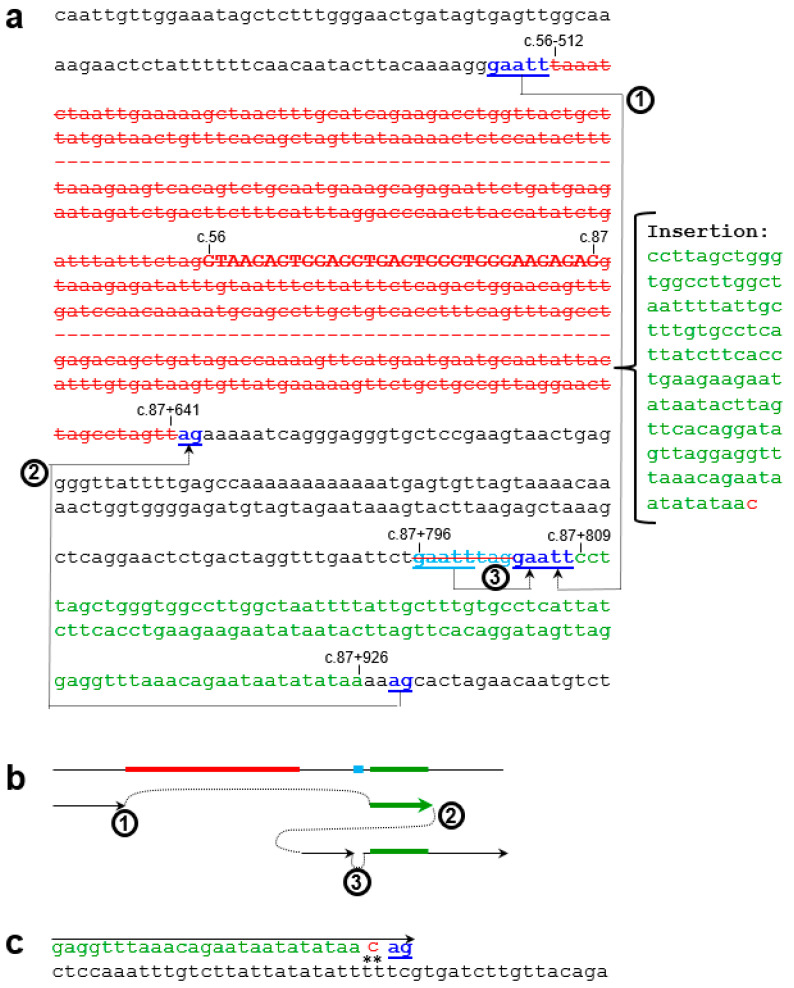
Illustration of the *SPINK1* complex rearrangement variant and proposed generative mechanism. (**a**) Sequence details of the complex rearrangement allele, shown on the *SPINK1* sense strand. *SPINK1* exon 2 (c.56 to c.87) is in bold uppercase; intronic sequences are in lowercase. The rearrangement consists of two components. The first is a 1185 bp deletion from c.56-512 to c.87+641 (highlighted in red and overbarred) accompanied by a 119 bp insertion. The inserted sequence corresponds to a 118 bp copy of a downstream region (c.87+809 to c.87+926; highlighted in green), followed by a single C. The second component is a small deletion of GAATTTAG (highlighted in cyan and overbarred). The rearrangement is attributed to serial replication slippage (SRS), involving two forward slippage events (steps ① and ③) and one backward slippage event (step ②). Relevant direct repeats (underlined and colored) are indicated. The positions of exon 2 boundaries, and of deleted and duplicated sequences, are labeled. Dashes represent omitted sequences. (**b**) Schematic of the three SRS steps, shown relative to the wild-type allele, with deleted and duplicated sequences in the same colors as in panel (**a**). (**c**) Illustration of translesion synthesis (TLS) potentially responsible for the single inserted C, presumed to result from synthesis across a damaged “tt” site (indicated by **).

Targeted NGS also indicated a heterozygous deletion of *SPINK1* exon 2 in the proband, as evidenced by an approximately 50% reduction in exon 2 coverage compared to two WT controls (Supplementary Figure S2). This heterozygous deletion was confirmed by QFM-PCR, in which the fluorescent peak corresponding to exon 2 was approximately 50% of that observed in the control sample (Figure 1c). To identify the deletion breakpoint, we performed long-range PCR using a forward primer located within the *SPINK1* promoter and a reverse primer located within intron 3. Sequencing of the aberrant shorter band (Figure 1d; Supplementary Figure S3) revealed a complex rearrangement comprising two components. The first is a 1185 bp deletion accompanied by a 119 bp insertion, and the second is a simple 8 bp deletion (Supplementary Figure S4; Figure 2). Notably, the deleted 1185 bp genomic segment includes the 32 bp exon 2. Loss of exon 2 from the *SPINK1* coding sequence would unequivocally result in a non-functional protein product. Specifically, this deletion removes coding nucleotides c.56 to c.87, thereby disrupting the reading frame and producing a severely truncated protein lacking essential functional domains. This variant has not been previously reported in the literature, is absent from gnomAD SVs v4.1.0, and is not registered in ClinVar (as of 16 June 2025).

In summary, the proband exhibited complete *SPINK1* deficiency due to compound heterozygosity for two complete LoF variants. In contrast, each parent is expected to retain approximately 50% of normal *SPINK1* function, consistent with simple heterozygosity for a complete LoF variant.

### 3.3. Generative Mechanisms and Nomenclature of the Novel Complex Rearrangement Variant

Close examination of the 119 bp insertion revealed that the first 118 bp was templated from c.87+809 to c.87+926, followed by a non-templated cytosine. This, together with the presence of short direct repeats at the deletion/insertion breakpoints (Figure 2a), suggests that this complex rearrangement can be attributed to replication-based mechanisms [30], the core of which involves serial replication slippage (SRS) or serial template switching [31,32]. Notably, the non-templated cytosine could also be adequately explained by the involvement of translesion synthesis (TLS) DNA polymerases in the process [33]. The key steps of the proposed mechanism are illustrated in Figure 2b,c. However, analysis using nBMST [29] did not reveal any specific non-B DNA motifs [34] that might underlie the formation of this novel *SPINK1* variant.

For this novel complex rearrangement variant, the presence of a non-templated single-nucleotide insertion following the 118 bp templated insertion made an exact HGVS-compliant description challenging. We therefore adopted a simplified notation to represent the variant: c.[56-512_87+641delins119bp;87+796_87+803del].

## 4. Discussion

In this study, we report a novel case whose phenotype closely matches that of the two previously described individuals with SIIEPI [19]. Shared features include early-onset steatorrhea, diffuse pancreatic lipomatosis, and the absence of extra-pancreatic manifestations. Consistent with these clinical observations, we identified a novel *SPINK1* genotype resulting in complete LoF, confirming a biallelic null genotype in this case. This finding reinforces the concept of SIIEPI as a distinct clinical entity caused by complete *SPINK1* deficiency [19]. It also supports the emerging view that humans with biallelic *SPINK1* LoF—functional “*SPINK1* knockouts”—are viable, and that SPINK1 function is confined largely, if not exclusively, to the exocrine pancreas.

Importantly, the two previously known SIIEPI cases were also reported by our group. Notably, these cases carried either gross deletion or insertion variants—types of variants that are difficult to detect by conventional Sanger sequencing alone. Indeed, all five currently reported CP- or SIIEPI-related gross *SPINK1* structural variants—including the complex rearrangement described in the present case (four deletions and one insertion)—[19,26,35] have been identified by our team using approaches capable of detecting structural variants, such as QFM-PCR followed by long-range PCR. This observation raises the possibility that these gross structural variants, as well as *SPINK1*-related SIIEPI, may be under recognized in clinical settings, especially if only standard sequencing methods are used without complementary CNV analysis or long-range PCR, let alone if only genotyping methods focusing on only known pathogenic or predisposing variants are being used.

*SPINK1*-related SIIEPI also warrants comparison with other genetic forms of early-onset exocrine pancreatic insufficiency, particularly those caused by pathogenic or predisposing variants in *CFTR* and *CEL*. Cystic fibrosis, for example, typically involves multi-organ dysfunction including chronic pulmonary disease [36], while *CEL*-associated MODY8 often presents with both endocrine and exocrine insufficiency [37]. By contrast, SIIEPI appears restricted to the exocrine pancreas. It is also distinct from syndromic forms of pancreatic insufficiency, such as Shwachman–Diamond syndrome and Johanson–Blizzard syndrome, which are associated with systemic or developmental abnormalities [38]. The absence of extra-pancreatic features and the consistent identification of biallelic *SPINK1* LoF variants across all reported cases strongly support its classification as a non-syndromic, pancreas-specific disorder. These distinctions are clinically important for accurate diagnosis and targeted genetic testing in infants presenting with isolated pancreatic insufficiency.

Beyond its clinical relevance, the novel complex variant described here also offers insights into mutational mechanisms affecting *SPINK1*. The rearrangement consists of a 1185 bp deletion, a 118 bp templated insertion, and an 8 bp microdeletion. The presence of short direct repeats at the breakpoints and the templated nature of the insertion are characteristic of replication-based mechanisms, such as SRS or serial template switching [30,39]. Additionally, the presence of a non-templated cytosine at the 3′ end of the insertion suggests the involvement of TLS DNA polymerases [40], which contribute to complex genomic rearrangements through low-fidelity gap-filling activity [33].

In conclusion, this study expands the mutational spectrum of *SPINK1* associated with complete LoF and provides further evidence that replication-based mechanisms can generate complex pathogenic rearrangements. Our findings also emphasize the clinical relevance of recognizing SIIEPI as a distinct, likely underdiagnosed pediatric disorder caused by biallelic *SPINK1* inactivation.

## Data Availability

The original contributions presented in this study are included in the article/Supplementary Materials. Further inquiries can be directed to the first or corresponding author.

## Supplementary Materials

The following supporting information can be downloaded at: https://www.mdpi.com/article/10.3390/genes16090998/s1, Figure S1: Sanger sequencing electropherogram showing the heterozygous 2 bp deletion in *SPINK1* exon 3, NM_001379610.1:c.180_181del, in the proband; Figure S2: Detection of *SPINK1* exon 2 deletion in the proband by targeted next-generation sequencing (NGS); Figure S3: Uncropped gel image corresponding to Figure 1d; Figure S4: Sanger sequencing electropherogram of the novel *SPINK1* complex rearrangement allele.

## Abbreviations

The following abbreviations are used in this manuscript:

LoF	Loss-of-function
SIIEPI	Severe infantile isolated exocrine pancreatic insufficiency
NGS	Next-generation sequencing
QFM-PCR	Quantitative fluorescent multiplex PCR
CP	Chronic pancreatitis
CNV	Copy number variant
WT	Wild-type
HGVS	The Human Genome Variation Society
gnomAD	The Genome Aggregation Database
nBMST	The non-B DNA Motif Search Tool
SRS	Serial replication slippage
TLS	Translesion synthesis
