# Supplementary material for: Compound Heterozygous Complete Loss-of-Function SPINK1 Variants as a Novel Cause of Severe Infantile Isolated Exocrine Pancreatic Insufficiency
Source: Genes (Basel). 2025 Aug 25;16(9):998. doi: 10.3390/genes16090998 (PMC12469571; doi:10.3390/genes16090998)
Supplement: Supplementary file 1 [file genes-16-00998-s001.zip › genes-3747420-supplementary.pdf]

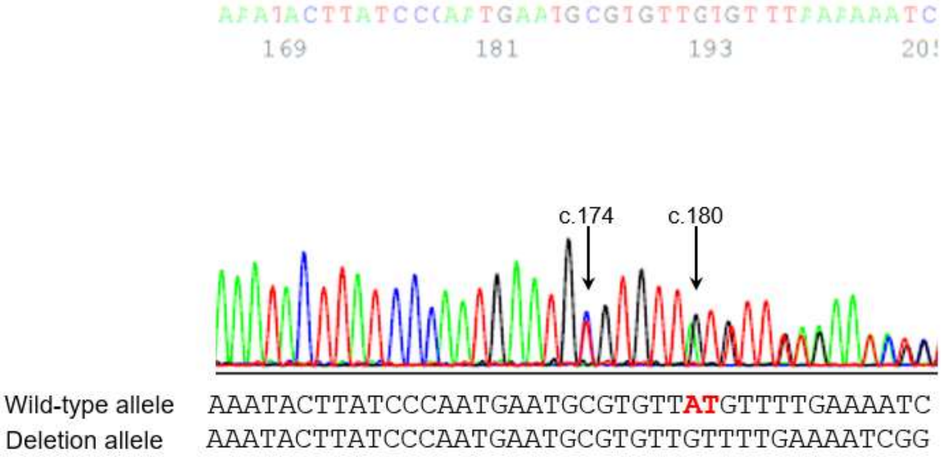

Figure S1. Sanger sequencing electropherogram showing the heterozygous 2-bp deletion in *SPINK1* exon 3, NM_001379610.1:c.180_181del, in the proband. The proband also carries a heterozygous synonymous variant, NM_001379610.1:c.174G>T (p.Cys36=), which was demonstrated to be in trans with the 2-bp deletion based on parental sequencing data. Sequences corresponding to the wild-type and deletion alleles are shown below the electropherogram. The deleted bases (AT) in the wild-type allele are highlighted in red.

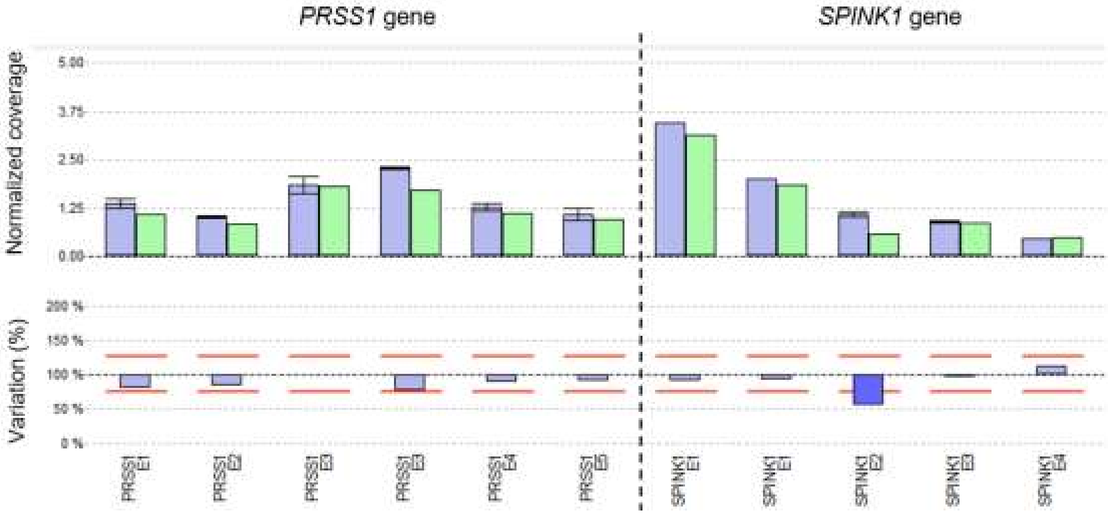

Figure S2. Detection of *SPINK1* exon 2 deletion in the proband by targeted next-generation sequencing (NGS). Data from one of the co-analyzed genes, *PRSS1*, are shown for comparison. The upper panel displays the normalized coverage of each amplicon in the proband (green) and the average normalized values from two wild-type controls (blue). The lower panel shows the relative coverage of each amplicon in the proband, expressed as a percentage of the control value. Amplicons with relative coverage below 75% or above 125% of the control average were interpreted as indicative of a deletion or duplication, respectively; these thresholds are indicated by red dashed lines. Note that *PRSS1* exon 3 (E3) and *SPINK*1 exon 1 (E1) were each targeted by two independent amplicons. The marked reduction in relative coverage for *SPINK1* exon 2 supports the presence of a heterozygous exon 2 deletion in the proband.

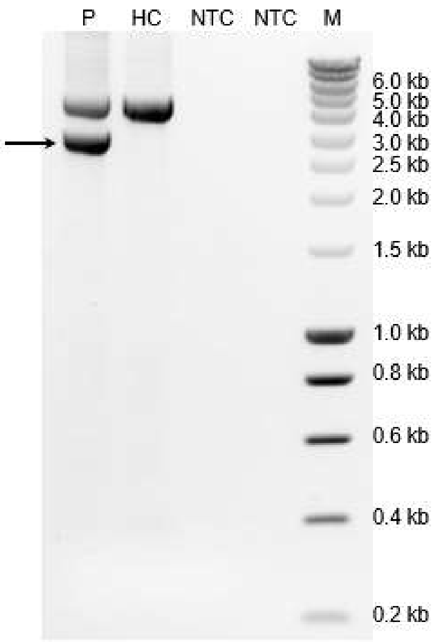

Figure S3. Uncropped gel image corresponding to Figure 1d. Long-range PCR products from the proband (P) and a healthy control (HC) are shown. The proband-specific ~3.0 kb band is indicated by the black arrow. NTC, no template control; M, DNA marker with sizes indicated in kilobases (kb).

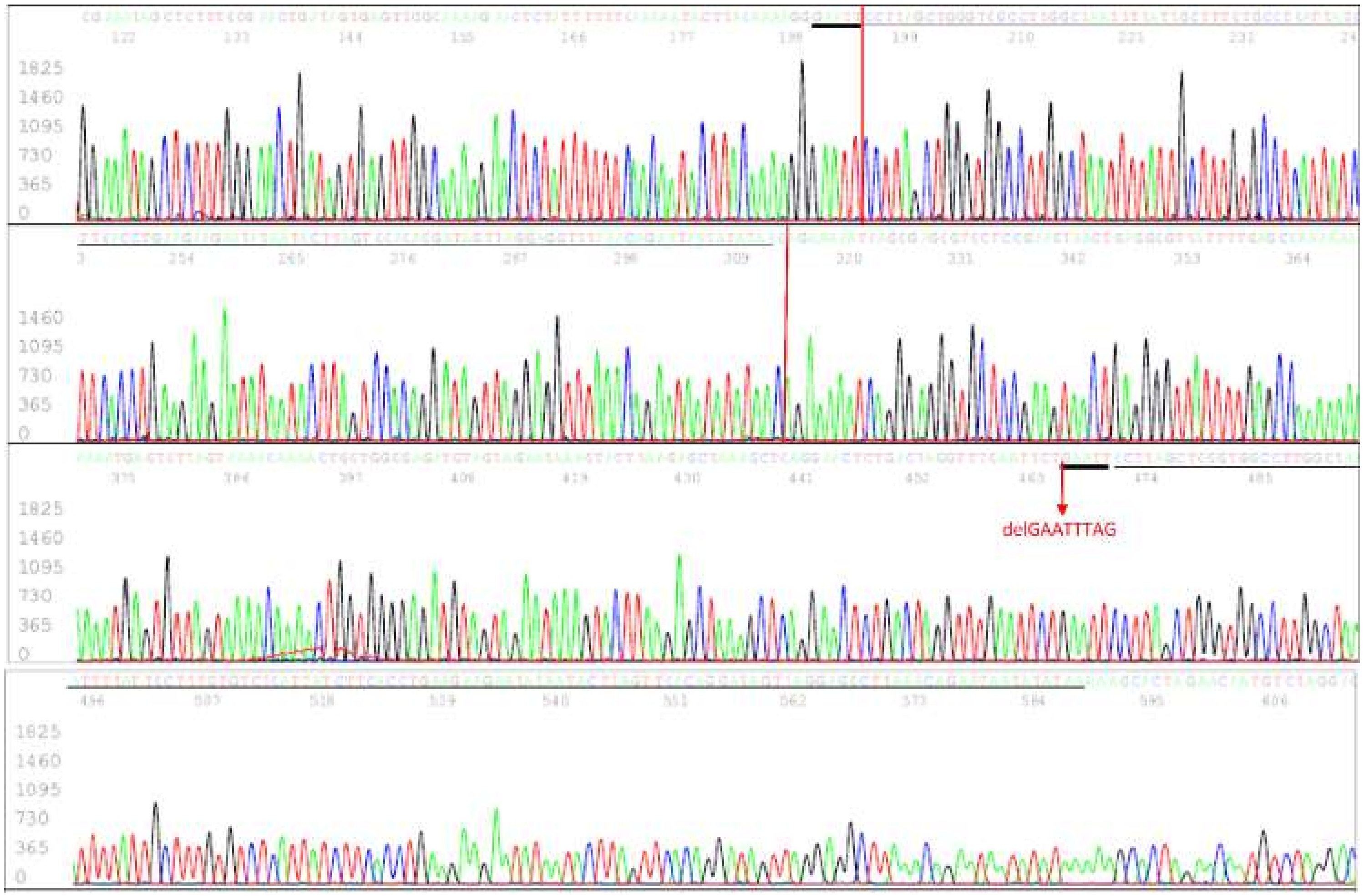

Supplementary Figure S4. Sanger sequencing electropherogram of the novel *SPINK1* complex rearrangement allele. Sequencing was performed on the aberrant long-range PCR product derived from the patient (see Figure 1d). The complex rearrangement consists of two components and is attributed to serial replication slippage. The first component involves a large 1,185-bp deletion spanning from c.56–512 to c.87+641 (encompassing the 32-bp exon 2), accompanied by a 119-bp insertion. The deletion boundaries are marked by vertical red lines. The inserted sequence, located between the two red lines, consists of a 118-bp copy of a downstream region (c.87+809 to c.87+926), indicated by a thin horizontal underline, followed by a single cytosine (C). The GAATT repeat motifs implicated in the initial slippage step are highlighted with bold underlines. The second component is a small deletion of GAATTTAG, indicated by a red arrow. See Figure 2 for further details on the mutant sequence and the proposed mechanism.
